# Affectivity as stance: multimodal stance-taking in audiovisual documentations of Polish and German parliamentary debates

**DOI:** 10.3389/fpsyg.2024.1467185

**Published:** 2024-11-27

**Authors:** Cornelia Müller, Maciej Karpiński, Clara Kindler-Mathôt, Katarzyna Klessa, Ewa Jarmołowicz-Nowikow, Jana Katharina Junge, Katerina Papadopoulou, Brygida Sawicka-Stępińska

**Affiliations:** ^1^Faculty of Social and Cultural Sciences, European University Viadrina, Frankfurt (Oder), Germany; ^2^Faculty of Modern Languages and Literature, Adam Mickiewicz University, Poznań, Poland

**Keywords:** multimodality, gesture, media analysis, political communication, discourse analysis, interaffectivity, multimodal discourse analysis, prosody analysis

## Abstract

This paper presents a media-aesthetic framework to study affectivity as a stance. This framework opens up a new perspective on multimodal affective stance-taking in the context of specific media ecologies. It exemplifies this new approach with case studies of the official audiovisual documentation of political debates in the German Bundestag and the Polish Sejm. This new approach addresses the intertwining of audiovisual multimodality with verbo-gestural expressivity (or the multimodality of speaking). Adopting a phenomenological position, we are interested in how the orchestration of the debates as audiovisual events moves the spectators. The concepts ‘Expressive Movement’ and ‘Dynamic Forms of Vitality’ serve as theoretical and methodological references to capture the affective dynamics of audiovisual debates and how these audiovisual images modulate the perceptions and experiences of the spectators. To illustrate and substantiate this approach for linguistic and media analyses of affective stance-taking, the paper outlines basic assumptions and methods. It offers two exemplary case studies from German and Polish parliamentary debates. It is concluded that bringing together media-aesthetic with linguistic analyses of multimodal communication and interaction provides not only a valid starting point for future research of multimodal stance-taking in different media ecologies but also allows researchers to address how and why spectators of audiovisual media performances are moved affectively.

## 1 Introduction

It is common practice in democratic societies to document the work of the parliament in an archive. Public debates of bills and budgets are one of the central activities of a parliament. Such archives function as “memories” of the parliament, but they also invite the public to participate in the debates of their elected representatives. How such political debates ‘move’ the spectators is the subject of a German–Polish research project, which explores multimodal affective stance-taking in the German Bundestag and the Polish Sejm. The project investigates audiovisual materials supplied by the respective archives of the budget and financial debates in the German and the Polish parliaments. Analyzing multimodal stance-taking in the context of audiovisual documentations of parliamentary debates requires a reflection on both the mediatized character of the material *and* the multimodal performance of the speakers and their engagement with the audience in parliament. Put differently, analyses face an intertwining of audiovisual multimodality and verbo-gestural expressivity (or what we consider: the multimodality of speaking). This has important consequences for the research into stance-taking.

Despite a rich and diverse literature on stance and stance-taking (Avdan, 2017; Biber and Finegan, 1989; Dancygier, 2012; Du Bois, 2004, 2007; Englebretson, 2007; Feyaerts et al., 2017; Haddington, 2006), the role of gesture, posture, and prosody, as aspects of this communicative activity, remain only scarcely studied. Although Du Bois’ paper on the stance triangle mentions gesture several times as one of the expressive forms that contribute to stance-taking (Du Bois, 2007, pp. 169, 171) and suggests speaking of stance-taking as an activity rather than of stance as a phenomenon of lexical semantics and text linguistics (Biber and Finegan, 1989), his insight did not foster a systematic inclusion of gesture, speech, and body movement in the study of stance-taking. Some of the few exceptions come from anthropology and conversation analysis (Goodwin et al., 2012; Ochs and Schieffelin, 1989) and gesture studies (Bressem and Müller, 2017; Ladewig, 2014, 2024; Müller and Speckmann, 2002), including shrugs and head tilts (Debras and Cienki, 2012). The role of prosody in stance-taking has been directly explored to a limited extent (Freeman, 2015a, 2015b). More recently, and in a plea to integrate conversation analytic and interactional perspectives on stance with the usage-based approach of cognitive linguistics, Feyaerts et al. (2017) lay out the significance of multimodal aspects of stance-taking specifically with regard to alignment. Cognitive linguist Dancygier (2012) introduces the notion of ‘stance-stacking’ to indicate that expressions of stance form constructions and often do not come alone, but build clusters. Dancygier’s study highlights the semantic complexity of stance and shows how language use in the media is permeated by visual modes of expression to a degree that they develop into multimodal constructions. Dancygier and Vandelanotte (2017) exemplify this with constructional analyses of internet memes. Taking an expressive stance is seen as *the* motivation behind making a meme.

The present paper presents a media-aesthetic framework to study affective multimodal stance-taking in audiovisual media. It offers a new view on embodied interaction in multimodal stance-taking, in both face-to-face interaction and when spectators engage with televised face-to-face interaction. This position draws on earlier study by Ochs and Schieffelin (1989) and Goodwin et al. (2012) and on media-aesthetic (Kappelhoff, 2015) and neo-phenomenological film theory (Sobchack, 1992). Ochs and Schieffelin (1989, p. 7) suggest that linguistic and discourse features “provide an affective frame for propositions encoded” and highlight the relevance of “gestural cues to provide interlocutors with critical information on which to base subsequent social actions.” Following up on this study, Goodwin et al. (2012) regard affective stance as “situated practice entailed in a speaker’s performance” and achieved “through intonation, gesture, and body posture” (Goodwin et al., 2012, p. 16).

The position advocated in this paper takes recourse to the idea of multimodal communication as a dynamic, intercorporeal, and hence *interaffective* process (Müller and Kappelhoff, 2018, chapters 1–5). Conceiving *affectivity as stance* thus addresses a basic form of human understanding that is grounded in an intercorporeal exchange of perceiving, experiencing, feeling. In such a phenomenological view, perceiving is experiencing the other as a *Moving Other*. Research on social interaction has shown that when people speak with one another, this being together is established through subtle forms of bodily synchronization (Kendon, 1972), it is maintained by mutual alignment (Feyaerts et al., 2017) and rhythm (Breyer et al., 2017). Phenomenology regards this intercorporeal being together as an *interaffective* dimension of experience (Fuchs, 2017). In order to grasp this interaffective dimension of experience in contexts of televised multimodal performances—as both a theoretical concept and empirical methodology, we work with the notions of ‘Expressive Movement’ (EM) and ‘Forms of Vitality’.

For the study of affective stance, we extend previous research on cinematic EMs as interaffective embodied experiences that ground metaphorical meaning-making in both face-to-face interaction and the process of viewing moving images (Horst et al., 2014; Kappelhoff and Müller, 2011; Müller and Kappelhoff, 2018). In our study, stance-taking is conceived as a public activity in which the complexities of multimodal stance expressions form multidimensional experiential gestalts that are permeated by affect—theoretically and analytically graspable as EM (Müller and Kappelhoff, 2018; chapters 2, 3, 6, 8).

To investigate the affectivity of multimodal stance-taking in audiovisual media we have conducted a research project on political debates in the German and the Polish parliaments, respectively. The paper presents insights and the first results from this larger undertaking. It begins with a brief outline of the basic assumptions of a media-aesthetic approach to affectivity as stance in section (2); section (3) introduces data and methods; (4) presents a comparative case study of multimodal affective stance-taking in parliamentary speeches; (5) offers a summary and a discussion; and (6) concludes the paper.

Why investigate affective stance-taking in parliamentary debates and speeches? Clearly, parliamentary speeches are meant not only to provide factual information but also to impress and convince addressees. Therefore, they are a rich resource for studying affective stance-taking. As official documentations of political debate, they are meant to be as ‘neutral’ or ‘objective’ as possible, so we would expect the audiovisual staging to be rather neutral and not to play a prominent role in the affective perception of the televised embodied performances. We will see whether this is in fact the case.

## 2 A media-aesthetic approach to affectivity as stance

This chapter draws on earlier study on Cinematic Metaphor (Kappelhoff and Müller, 2011; Müller and Kappelhoff, 2018). Against the backdrop of this transdisciplinary approach, the present paper brings together film theory and linguistics to analyze televised forms of face-to-face interaction. The theoretical positions outlined in this section sketch a media-aesthetic framework for the multimodal analysis of affective stance in audiovisual documentations of political debates. Starting from the expressive character of multimodal communication, these basic theoretical assumptions lay the ground for media-aesthetic and linguistic methods for researching multimodal affective stance-taking. These theoretical points are particularly relevant here: affectivity as a dynamic form of vitality (2.1), Expressive movement as multidimensional experiential gestalt (2.2), and affective stance as a media phenomenon (2.3).

### 2.1 Affectivity as a dynamic form of vitality

In his study on the interpersonal world of the infant, Daniel Stern describes the attunement of parent and newborn child as a dance (Stern, 1985). Dancing together means moving together. It involves feeling the movement dynamics of the other person in one’s own body. In his later study, Stern extends his observations from early child development to the “experience of vitality” as a dynamic form that “permeates daily life, psychology, psychotherapy, and the arts” (Stern, 2010, p. 3). Stern argues that “We naturally experience people in terms of their vitality” and that also the “Time-based arts, namely music, dance, theater, and cinema […], move us by the expressions of vitality that resonate in us.” (Stern, 2010, p. 4). Drawing on Stern’s approach, we consider affectivity as a *quality of movement* where ‘movement’ includes body movement, speech movement, and the movement of audiovisual images. Rather than conceiving of affect as an expression of some inner state discernible as facial expression (Ekman, 1972, 1992), affectivity is described as a form of experienced vitality, a dynamically *unfolding temporal contour*. Stern describes them as a crucial dimension of being:

They are the felt experience of force – in movement – with a temporal contour, and a sense of aliveness, of going somewhere. They do not belong to any particular content. They are more form than content. They concern the “How,” the manner, and the style, not the “What” or the “Why” (Stern, 2010, p. 7).

**Figure 1:**
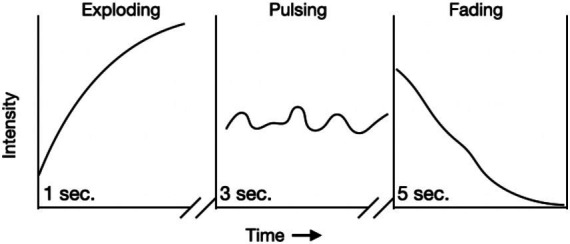
Stern’s dynamic forms of vitality, three possible vitality forms (p8).

Stern directs our attention to a group of words that describe such forms of vitality as felt experiences, exploding, pulsing, or fading describes forms of vitality (Figure 1). An exploding form of vitality shows a steeply rising curve, a pulsing one, and a series of small curves on a steady horizontal level; a fading vitality is a continuously declining contour. Stern illustrates the “felt experience of force in movement as a temporal contour” as graphs of time and intensity (Figure 1).

Forms of vitality concern the dynamic expressive qualities of movement. They address how a movement is performed. They are bound to movement and time, and they are modality-independent. Here is where Stern connects bodily forms of vitality with time-based arts.

The same is true for the time-based arts. The dynamic flow of music (sound in motion), dance, theater, and cinema sweeps us up at moments and then releases us, only to sweep us up again quickly just downstream (Stern, 2010, p. 6).

These commonalities between the time-based arts and body movements are essential for a media-aesthetic approach multimodal affective stance. They allow us to account for affectivity as a dynamic form of vitality that is expressed in the movement dynamics of both the delivered speech and the audiovisual documentation of it.

### 2.2 Expressive movement as multidimensional experiential gestalt

How precisely are these movement contours perceived? In order to understand this, we need to consider the perception of body movements further, and here is where the concept of Expressive Movement comes in (Kappelhoff, 2004, part 1; Kappelhoff, 2018b; Müller and Kappelhoff, 2018, chapter 9). It concerns a specific understanding of body movements. The concept is historically rooted in early 20th-century discussions in philosophical anthropology, psychology, and linguistics. Expressive Movement offers an alternative perspective on the idea of expression as an involuntary, outer index of a subject’s inner disposition. Scholars like Bühler, Plessner, and Wundt shared the assumption of expressive behavior as an interplay of affective exchanges of intensity between living beings. In Plessner’s philosophical anthropology, for example, Expressive Movements are not expressions of inner states, but a form of behavior that synchronizes an organism with its environment. Body movements evolve as a wholeness, a movement gestalt, be they affective or affectless:

These wholenesses belong to the organism through its relation to the environment, […] As a result, the movement shapes are pictorial, even stretched out over a certain duration of time […] Grasping, fleeing, repelling, seeking, but also the “affectless” forms such as walking, flying, swimming […] represent such movement-images (Plessner, 1982 [1925], p. 78).

The Expressive Movements unfold as movement shapes, which become movement gestalts or movement images in the process of perception. Moreover, following Stern, body movements (including speaking) and audiovisual images are equally time-based forms of expressions, they not only unfold as a specific dynamic contour of movement but also they both become movement images in the perception of a co-participant or a spectator at a screen (see Kappelhoff’s notion of “Cinematic Expressive Movement,” Kappelhoff, 2004, part 1; Kappelhoff, 2008; Kappelhoff, 2018b; Müller and Kappelhoff, 2018). Expressive Movements are thus gestalts that emerge in a process of movement experience. They are orchestrated along multiple dimensions. For kinesic Expressive Movements, this includes speech, gesture, posture, gaze, and head movement; for cinematic Expressive Movements, this involves all the aspects of cinematic orchestration of audiovisual images, such as camera angle, camera movement, montage, sound, and mise-en-scène. The concept of Expressive Movement thus suggests a shared understanding of body movements and cinematic movement images.

The affective dimension of Expressive Movements *is* their quality of movement. This position aligns with Merleau-Ponty’s phenomenology, where gestures of anger or threat do not express an inner emotion, but *are* anger or threat: “Faced with an angry or threatening gesture, I do not see anger or a threatening attitude as a psychic fact hidden behind the gesture, I read anger in it. The gesture does not make me think of anger, it IS anger itself” (Merleau-Ponty, 2005 [1945], p. 184). Affectivity in this perspective is intercorporeal and interaffective, it is a form of embodied understanding. Expressive Movements are thus conceived as ‘multidimensional experiential gestalts’ that modulate and ground embodied processes of meaning-making affectively. Expressive Movements can be body movements or audiovisual movement images (Müller and Kappelhoff, 2018, chapters 1, 8, 9). In mediatized audiovisual documentations of political speeches, the dynamic unfolding and the internal orchestration of Expressive Movements frame the multimodality of speaking.

### 2.3 Affective stance in mediatized political debates

What are the consequences of such a position for an analysis of stance-taking in mediatized political debates? First, it underlines DuBois’ understanding of stance as an inherently multimodal and undeniably complex action. Du Bois defines stance.

“as a public act by a social actor, achieved dialogically through overt communicative means (language, gesture, and other symbolic forms), through which social actors simultaneously evaluate objects, position subjects (themselves and others), and align with other subjects, with respect to any salient dimension of value in the sociocultural field” (Du Bois, 2007, p. 169).

Second, it supports the idea of stance-taking as an activity rather than a phenomenon of lexical semantics and text linguistics (Biber and Finegan, 1989). It also underlines positions by Ochs and Schieffelin as well as by the Goodwins, who consider stance as a joint doing brought about by cooperating participants in an interaction (Goodwin, 2017; Goodwin et al., 2012; Ochs and Schieffelin, 1989).

**Figure 2:**
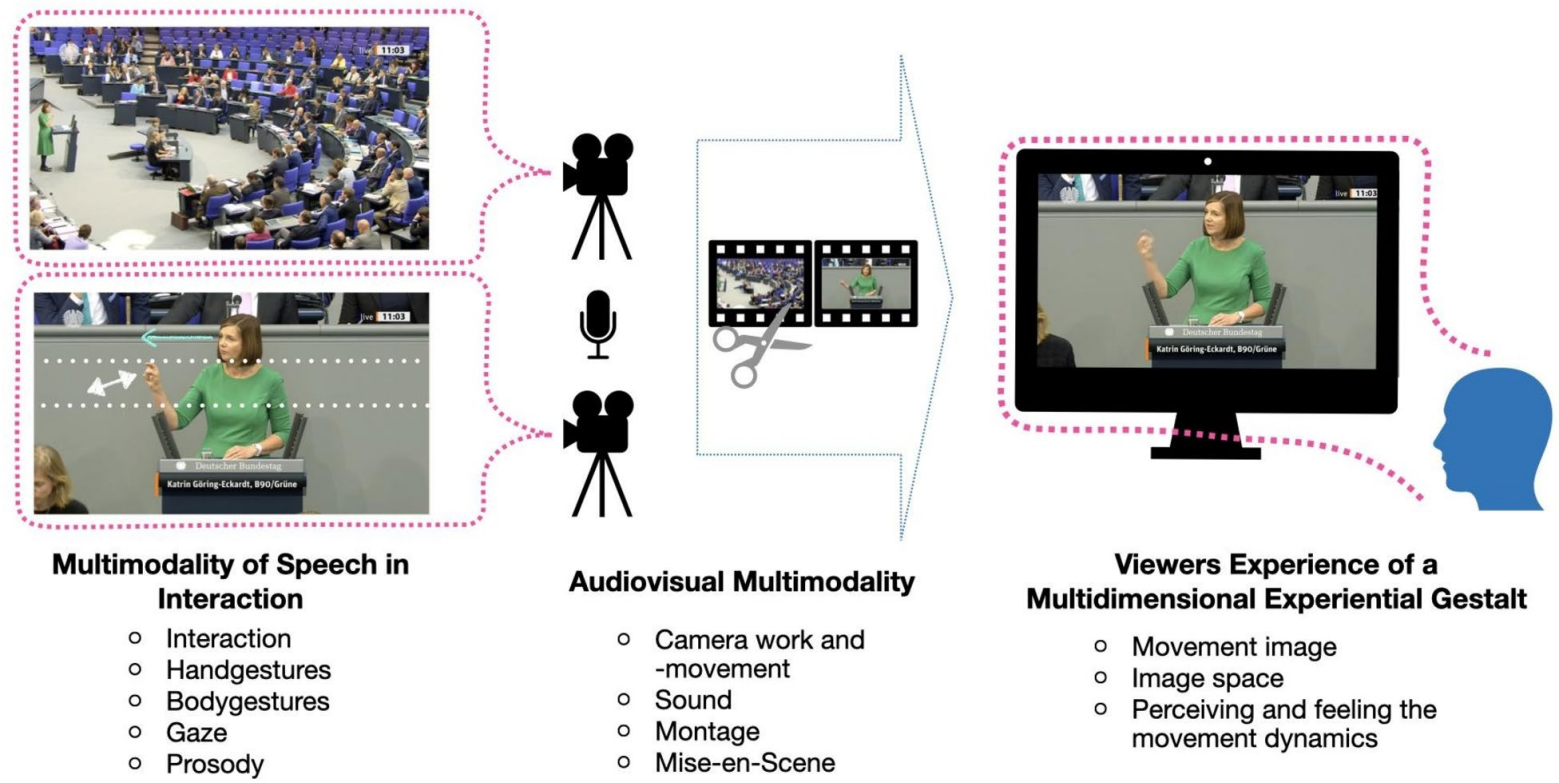
Two forms of multimodality forming the multidimensional experiential gestalt in the viewers’ perception. Recordings from parliamentary debates are sourced from Deutscher Bundestag.

But why consider the audiovisual orchestration of televised multimodal stance-taking? One could simply ignore the character of the audiovisual recording and directly analyze the verbo-gestural or multimodal performance of the speaker. Doing so would analyze what the analysts were able to see and leave aside movements not visible on the film image. Multimodal stance-taking would be reconstructed as a speaker’s process only. However, aside from competing theoretical positions concerning the nature of speaking as an individual or an interactive process, this kind of analysis would give no clue as to how the political speeches are perceived by the audiences watching them on their screens. It would neglect the question of how audiovisual images affect their audiences so deeply—a question rendered even more acute since the popularity of TikTok videos. One answer to this has been formulated by media-aesthetic film theory: cinematic staging affects viewers bodily (Kappelhoff, 2004, 2018a, 2018b; Sobchack, 1992) (Figure 2).

Understanding film is not a cognitive puzzle of finding a storyline, it is experiencing cinematic movement images, and it is feeling the film. Neo-phenomenological film theorist Vivian Sobchack argues that it is spectators who lend the film their body. The film—as with all audiovisual images—materializes in the bodily sensations of a spectator:

“Following Sobchack “[…] any movement occurring in the composition unfolds outside the physical film material, outside the screen. It is materialized as bodily sensation of/in the viewer, it is embodied by that viewer (Sobchack, 1992, p. 9) […] The Expressive Movement articulated in the medium of cinematic movement-images gains its affective reality as viewer’s physical sensations. […] And it is exactly in this cinematic dimension of movement, through which all constitution of meaning goes, where Sobchack finds the basis for the intersubjective dimension of cinema”: (Müller and Kappelhoff, 2018, p. 64f).

This is how cinematic expressivity frames political debates affectively: viewers experience audiovisual images as bodily experience. If we connect this with Stern’s idea of forms of vitality as characterizing face-to-face interaction as much as the cinema, music, or theater, then we get a feeling for the fundamental impact cinematic staging of affectivity may have on how multimodal speeches are felt and understood by spectators at their screens, namely as stance.

This is why we consider mediatized affective stance to be orchestrated by cinematic Expressive Movements. They modulate the audiovisual-represented verbo-gestural Expressive Movements of a given speaker, thereby mobilizing the affective experience of the viewers. As a consequence, we distinguish two aspects of multimodality in our analysis: the multimodality of speaking and audiovisual multimodality. We look at how they are intertwined, and how the orchestration of the audiovisual image (e.g., a close-up versus a wide-angle shot) frames the perception of the spectators of the political speech on screen.

## 3 Data and methods

### 3.1 The data

The full corpus of the project includes speeches from the 2019 and 2020 financial debates of the *Polish Sejm* and the *German Bundestag*. Audiovisual recordings were obtained from the official archives of both parliaments^1^^,^ ^2^^,^ ^3^. These recordings are freely accessible to any interested citizen.

#### 3.1.1 The recordings

The Polish speeches were recorded on 8 January 2020 and the German ones on 10 and 11 September 2019. The German material consists of two debates (15 plus 23 speeches) with a total of 38 speeches of varying lengths (02:33–35:08 min). The Polish recordings contain 69 significantly shorter speeches (00:51–26:08 min). However, in total the length of the recorded material did not differ significantly: 323 min for the German and 339 min for the Polish videos. Each debate begins with the longest speech and ends with the shortest. In both parliaments, time limits are assigned beforehand. Speech slots are distributed based on political position and party affiliation. Each speaker is summoned by the President of the parliament and walks from their place to the rostrum. In both settings, the seat of the President and the seats for higher-ranking officials are positioned behind the speaker. The audience and other parties are arranged in a crescent tribune facing the speaker. In the *Bundestag*, camera shots show the speaker as well as the audience from the upper left, upper right, and upper front angles. In the *Sejm*, speakers are typically shown using a distant wide-angle camera when approaching the rostrum and a medium shot during the speech. In both parliaments, the footage is usually interleaved with the views of the audience, especially at the beginning and at the end of speeches and sometimes during applause.

#### 3.1.2 Data storage

For data storage, exchange, and annotation management, we used the Corpus Mini client–server database system (Karpiński and Klessa, 2018, 2021, pp. 83–93). The Corpus Mini management system facilitates the monitoring of annotation and analysis workflow in multimodal corpora containing various kinds of multimedia files, annotations, and metadata materials. The present dataset includes. WAV and. MP4 files along with annotation files in the formats supported by Praat (Boersma and Weenink, 1992–2024), ELAN (Wittenburg et al., 2006), and Annotation Pro (Klessa et al., 2013). In Corpus Mini, each collection of files related to one recorded situation is treated as one data bundle named a recording session. Interoperability between annotation formats is ensured due to the implemented import–export options, which are crucial for the present context that requires the inspection of multimodal interactions based on measurements and annotation mining involving multiple parameters and annotation layers.

### 3.2 Methods

The methods outlined in this section bring together an empirical media-aesthetic (descriptive, phenomenological) approach with an instrumental analysis of speech prosody and a linguistic analysis of gestural performance.

They are an extension of the film-analytical and linguistic methods initially developed for the analysis of Cinematic Metaphor (CinMet). CinMet.

“[…] addresses the temporality of meaning-making as a specific mode of perceiving, sensing, and feeling and offers different forms of visualizations of this temporal affectivity and the dynamics of […] meaning[−making]. Our starting point is the temporality of experiencing which characterizes film-viewing as much as face-to-face interaction.” (Müller and Kappelhoff, 2018, p. 227).

Note that the term ‘cinematic’ refers here to the kinematic or ‘movement’ nature of both—face-to-face interaction as ‘dance’ in Stern’s sense *and* film-viewing as an interactive process between ‘movement-images’ and spectators (Müller and Kappelhoff, 2018). Based on this earlier work on affectivity and multimodality, we approach stance-taking as it manifests itself in Expressive Movements. Again, we draw here on earlier study (eMAEX, electronically mediated analysis of Expressive Movements Kappelhoff et al., 2016; Müller and Kappelhoff, 2018, Appendix). For the analysis of moments of affective stance-taking in parliamentary speeches, we have focused on Expressive Movements that display a high affective engagement. These were described in terms of the movement qualities or vitality contours of the perceived movement gestalt and their multimodal orchestration.

To ensure intersubjective accuracy, analyses were carried out in independent tandems of German and Polish researchers. To complement the intersubjectively qualified analyses, instrumental measures of the basic prosodic properties were carried out. A moving time window approach was applied to capture changes in pitch frequency, intensity, speech rate (based on segmental duration), and normalized PVI. Time-group analysis was carried out for interpausal units identified in the realization of the EM.

### 3.3 Analytic procedure: affectivity as a form of stance-taking

**Figure 3:**
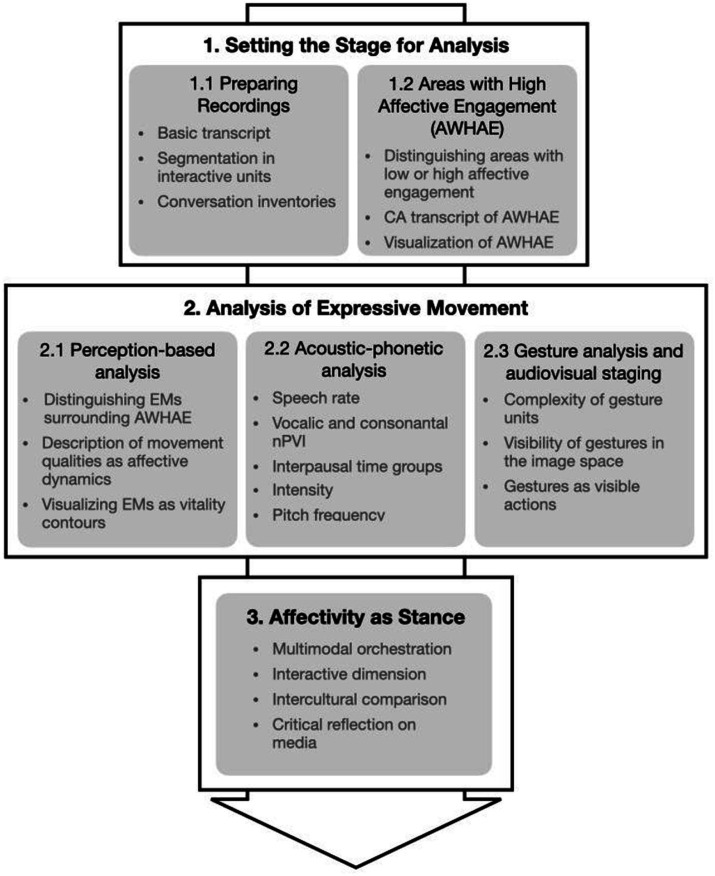
Analytic procedure: (1) setting the stage for analysis, (2) analysis of expressive movement, and (3) describing affectivity as stance.

Figure 3 shows an overview of the analytic procedure. The procedure falls into three larger steps: (1) setting the stage for analysis, (2) analysis of EMs, and (3) analysis of affectivity as stance.

#### 3.3.1 Setting the stage for analysis

Analysis begins with a first preparation of the recordings (Figure 3, 1.1): It includes a transcription in the annotation software ELAN (Wittenburg et al., 2006) and Annotation Pro and the segmentation of the speeches into interactive units. Interactive units are bounded either by applause or by audible or visible interjections that receive a reaction from the speaker (the reaction can be minor such as raising the voice or a change in the flow of speech). In addition, making conversation inventories provides a first descriptive understanding of ‘what’s going on’ in these interactive units.

Then, Areas with High Affective Engagement (AWHAE) are analyzed (Figure 3, 1.2), starting with annotating affective peaks and then distinguishing between areas with low and high affective engagement. The “identification” of the peaks does not imply an adding up of individual expressive modalities but rather follows the approach of temporally unfolding multidimensional expressive forms. They emerge as clearly perceptible changes in intensity from the so-called baseline, i.e., the basic rhythm of the speech, as, e.g., explosive, steadily rising, or suddenly falling qualities. Baseline refers to the specific, individually characteristic speech style—or the multimodal dynamics of speech, from which the peak stands out. Affective peaks and baselines can only be understood as interrelated qualities of multimodally orchestrated speech. To prepare for micro-analysis of gestures and speech, a conversation analytic transcription of the AWHAE is made.

#### 3.3.2 Analysis of EM

In the second step, the analysis of EMs begins. The concept of Expressive Movement serves as an analytical tool to capture an affective stance from the point of view of the perception of the viewers and listeners of the speeches on a screen. This means we consider the intertwining of the multimodality of speaking (as orchestration of speech and gesture) with the staging of it as audiovisual performance (audiovisual multimodality). The analysis of EMs in these speeches falls into three further analytic aspects: a perception-based analysis (2.1), an acoustic-phonetic analysis (2.2), and an analysis of gestural performance and audiovisual staging (2.3).

##### 3.3.2.1 Perception-based analysis

In the perception-based descriptive analysis of EMs, the multimodal interplay of gestures, speech, and audience interventions in its dynamic affective unfolding is at stake. To capture the temporal unfolding of the affective dynamics, AWHAE are depicted as red triangles and blocks (using Inkscape as a tool). The EMs in which these affective peaks are embedded are shown as contours section 4.1).

**Figure 4:**
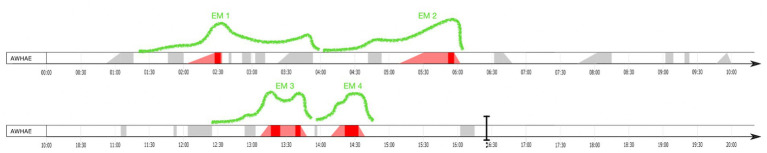
Bundestag speech: affective peaks along the speech (high affective engagement in red, lower in gray).

Sometimes EMs may include two or more affective peaks, sometimes only one. Their different shapes are the different forms of perceived and experienced affectivity. In the next analytic step, these affective qualities of the EMs are described in terms of their movement qualities. We use Stern’s list of adjectives as inspiration for the description (Greifenstein, 2020).

As in Stern’s descriptions of vitality contours, the affective dynamics of EMs are variable. Expressivity changes as the speeches unfold and evolve in their interplay with the audience and depending on the camera angle and montage (the perceived audiovisual expressivity differs according to whether we see a speaker close up or in a wide-angle shot). It is this kind of affective expressivity that we try to capture and describe from various angles and in a combination of visualization and subjective verbal description. We seek to capture stance as affectivity as embodied in these multidimensional experiential gestalts *as the audience on the screens* perceives them. Agreement between researchers was extremely high—although we did not perform interrater reliability tests.

##### 3.3.2.2 Prosody analysis

The next step addresses the dynamic contours of EMs with an instrumental analysis of the prosodic features. This analysis provides an independent source of ‘evidence’ for the perception analyses of the EMs.

Emotional prosody is placed very close to gestures in terms of expressive potential and conveyed meanings (Gibbon, 2005, 2011). Similarly to gestures, emotional prosody in adults may be stylized, modulated, or filtered by socio-cultural factors (Abelin and Allwood, 2000; Scherer, 2003; Scherer et al., 2011). In the study of emotional prosody, the acoustic-phonetic approach aims to identify and instrumentally measure acoustic correlates of prosodic features associated with emotionality. The results are often juxtaposed or coupled with perception-based experiments or expert listening judgments to test their relevance to human perception and the context of language communication. In the present study, several prosodic features have been identified in the expression of attitudes and emotions. We measured, in particular, changes in speech rate, normalized PVI, basic TGA parameters (slope and intercept), intensity, and pitch frequency.

Instrumental measurements of a selection of prosodic parameters pertaining to pitch, duration, and intensity were conducted using Praat (Boersma and Weenink, 1992–2024) and Annotation Pro (Klessa et al., 2013). Pitch frequency (in semitones, relative to the base frequency of 1 Hz) and intensity (in dB) were quantified utilizing respective Praat algorithms, wherein pitch extraction parameters were tailored to the speaker’s gender. The resultant pitch and intensity tiers were then imported into Annotation Pro and utilized as input values for a moving average algorithm (cf. Karpiński et al., 2014), employing a 2-s time window with a 1-s time step. The moving average approach served as a means of smoothing the raw data to mitigate random fluctuations or abrupt changes in the measured values. To capture local variations in segment duration, normalized Pairwise Variability Index (nPVI) (Grabe and Low, 2002) was automatically computed for vowels and consonants separately, employing a plug-in for Annotation Pro (Klessa, 2016). Furthermore, we investigated syllable timing variability within pause-delimited stretches of speech (interpausal time groups), following the time group analysis (TGA) approach proposed by Gibbon (2013). We used the Annotation Pro implementation of TGA (Klessa and Gibbon, 2014). The TGA method uses the linear regression function of syllable durations, mainly for the regression slope values, as a first approximation to examining speech acceleration and deceleration patterns. The mean slope values calculated over large data sets were observed to serve as a potential indicator of speaking style for several languages (e.g., Gibbon et al., 2014). Discussing our results, we refer also to the baseline values of the above variables, i.e., the values measured in a selected stretch of speech (*ca*. 12 s) not belonging to any EM and recognized by experts as typical of a given speaker, emotionally neutral.

All the semi-automatic measurements were scrutinized for potential issues that might have resulted from the features of the recordings (e.g., noise and voices from other speakers). As the authors did not control the recording conditions, measurements may be, in general, less reliable, especially in the case of intensity where the distance from the microphone and speaker’s orientation may bring in significant changes in the signal level. Furthermore, popular audio processing techniques such as noise gates and compression may distort the image of the original signal’s dynamics. It is worth noting, though, that other prosodic features used in the study (duration and pitch frequency) are less sensitive to these aspects of recording quality, and that time-related analyses underwent double revision.

Obviously, there is more to how multimodal EMs are orchestrated than speech prosody and gesture, but in the moments of high affectivity that we are particularly interested in, these two appear to play an important role. In particular, when considering the audiovisual staging of the speeches, it appears that the complexity and visibility of gestural movements feature prominently in how affectivity as stance becomes a felt perception of political performance for online spectators of such events.

##### 3.3.2.3 Gesture analysis

**Figure 5:**
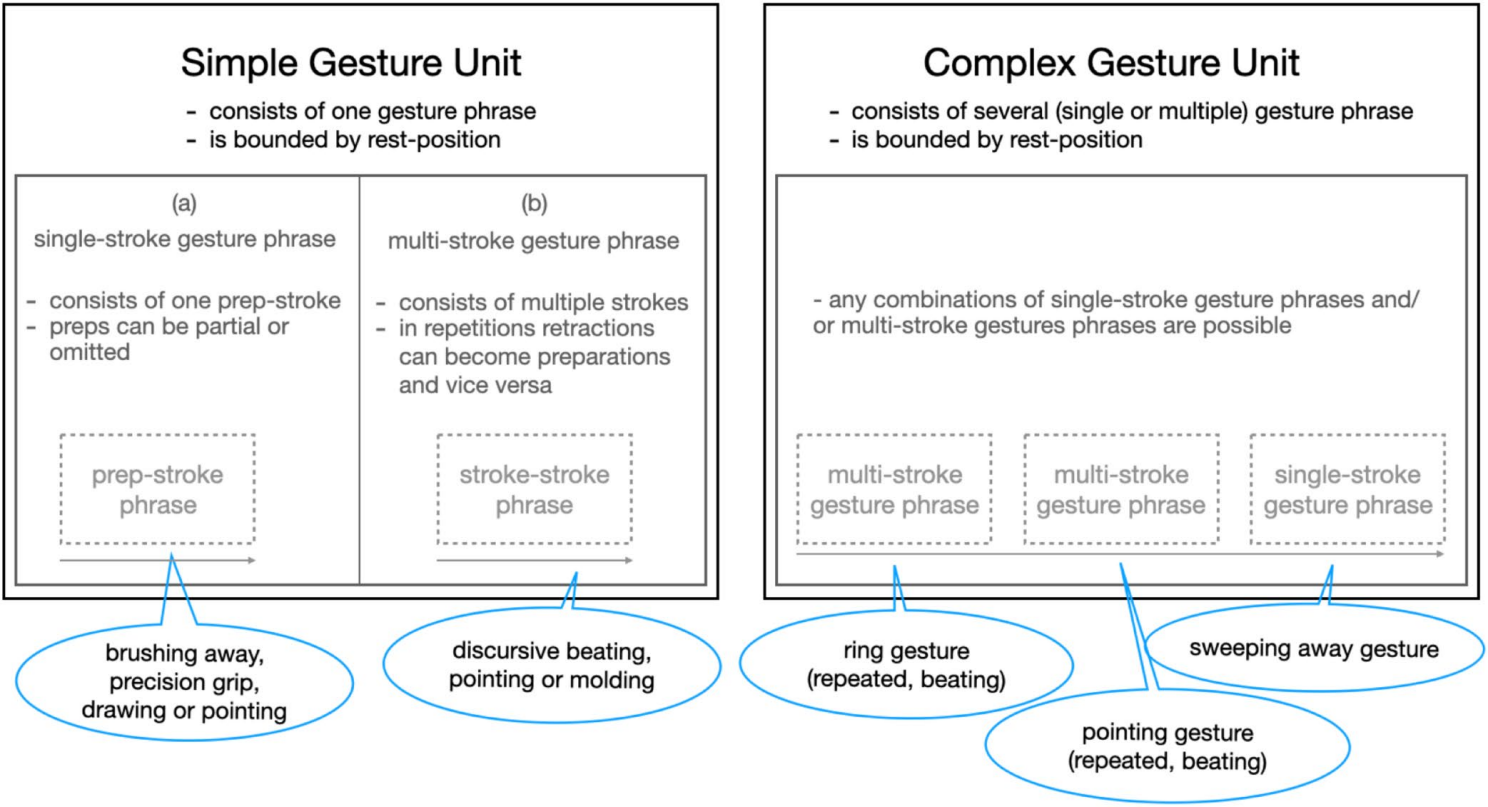
Simple and complex gesture units.

Gesture analysis takes into account how gestures are performed during EMs. Different aspects of gestural performance may become relevant, and the micro-analysis of gestures is adapted accordingly (Müller, 2024). In the micro-analysis carried out for this paper, we consider the complexity, the visibility of gestural movements, and their character as visible actions in the Kendonian sense (Kendon, 2004) as they unfold over the course of an EM. In terms of their complexity, gestures are considered as temporal forms, which may be segmented into gestural units, phrases, and phases (Kendon, 2004, chapter 7) (Figure 5). Gesture units appear in simple and complex forms and are delimited by rest positions (Kendon, 2004, p. 111ff). Simple and complex gesture units differ regarding the number of gestural phrases that unfold along such a gestural unit of expression. Kendon’s notion of gesture phrase includes preparation and stroke (cf. Kendon, 2004, p. 111ff.). Occasionally, a simple gesture unit may also be internally complex, namely when a gesture stroke unfolds with a complex movement pattern.

In addition to the unfolding complexity of gestural movements, we also considered the visibility of the gestural performance. Here, we considered in particular the height of their execution in gesture space. In gesture studies, McNeill’s (1992, p. 89) systematics of different gesture spaces is an important analytical reference point. They concern, for example, the intersubjectivity of the gestural movements: a gesture that is placed high up in the gesture space moves into the central field of visual attention of co-participants. Such a gesture is foregrounded because it cannot be overlooked by attending partners in a conversation (Müller, 2008; Müller and Tag, 2010). To account for the visibility of gestures in moments of high affective engagement, we considered the gestural movement in its path and location through the gesture space.

As the execution of a gesture often involves the movement of the hand through several gestural zones, we took into account only the moment of the gesture where the hand is furthest from the starting point. The reference point for describing the height of gesture execution was the speaker’s body. Gestures executed at the level of the abdomen were marked as 1, at the lower chest level 2, at the upper chest level 3, at the shoulder level 4, at the head level 5, and above the head 6.

**Figure 6:**
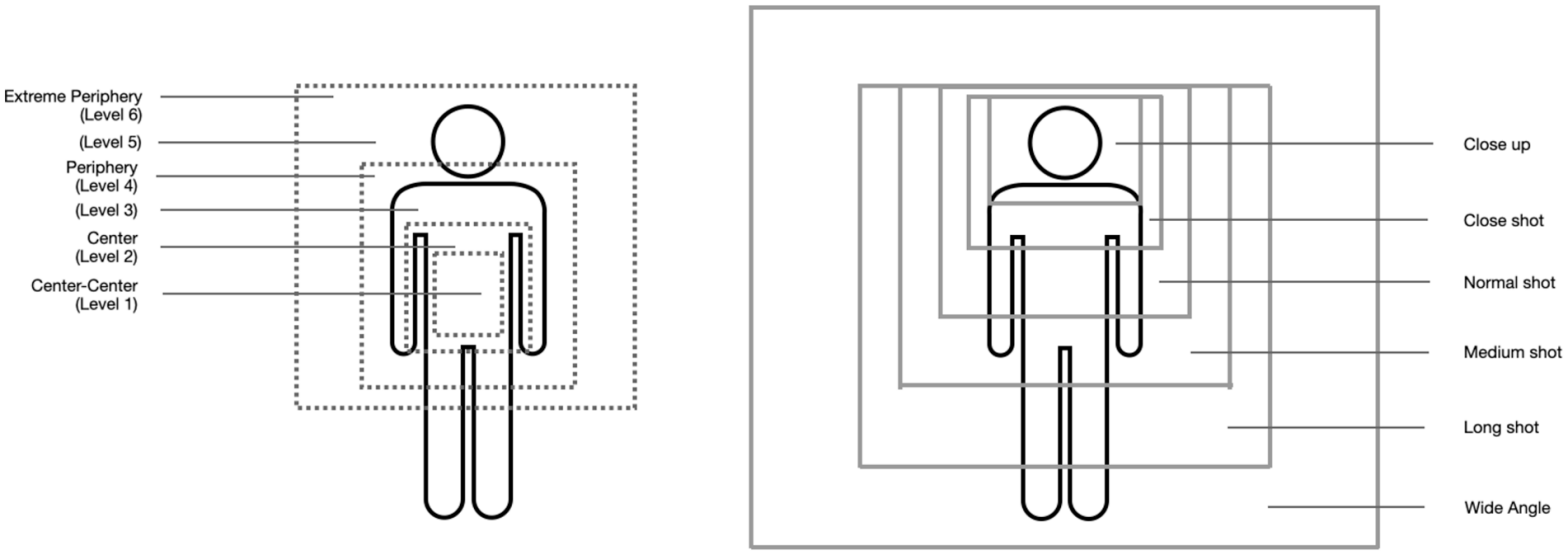
Gesture space and image space.

In particular, the visibility of the gestures in audiovisual documentations depends crucially upon another factor: the orchestration of the audiovisual image space. Here is where audiovisual multimodality frames and shapes what of the gestural performances the spectators are able to see. That is why the EM analysis concerns the intertwining of the gestural performance with the orchestration of the audiovisual movement-image. We distinguish the following shot sizes: close-up, close shot, normal shot, medium shot, long shot, and wide angle (Figure 6, right).

Combining instrumental and descriptive analyses of EMs offers an alternative window on what otherwise could be easily dismissed as ‘only’ subjectively perceived movement gestalts. Because prosody is a movement that makes a sound (Gibbon, 2005, 2011), the analysis of prosody is an interesting complement to the perception-based description of multimodal EMs, as both speech prosody and gesturing are body movements that contribute to a multimodal, multidimensional gestalt that we conceive here as one EM.

Describing affectivity in terms of stance, box 3 of Figure 3, is discussed in detail and with reference to the analysis of expressive movements in the following and in section 5.

## 4 Analyzing affectivity as stance in parliamentary debates

For this article, a close analysis of one speech from each of the German and the Polish debates serves as case studies to exemplify the media-aesthetic approach to multimodal affective stance-taking. This analysis exemplifies how multimodal EMs may serve as a frame for the investigation of affective multimodal stance-taking in recordings of parliamentary speeches. In the following, a detailed analysis of one expressive movement with an area of high affective engagement from the Polish and the German material is offered. We begin with a perception-based, descriptive account of the affective qualities of the EM, complement this with an acoustic-phonetic analysis of the parts of the speech that entail this EM, and end with a micro-analysis of the gestural performances and the audiovisual staging of the two EMs.

### 4.1 Bundestag: the speech and speaker

The speech is given by Katrin Göring-Eckardt (KAGE), the parliamentary leader of the *Green Party* (*Bündnis 90/Die Grünen*). Her speech has a total length of 16:13 min. It is the 6th speech of the second day of the financial debate with a total of 23 speakers and a length of 3:45 h altogether. The budgetary and the financial debates are part of the regular agenda of the chancellor. Chairpersons of the opposition challenge the budget of the government, whereas the chancellor and other members of the government hold speeches to defend it.

### 4.2 Sejm: the speech and speaker

This speech is delivered by Dobromir Sośnierz (DOSO), a representative from the right-wing conservative party, *Liberty and Independence Confederation* (*Konfederacja Wolność i Niepodległość*). Dobromir Sośnierz’s speech lasted a total of 1 min and 8 s. It was the 70th speech in the entire budgetary debate, which featured a total of 135 speeches. The debate took place in a single day.

Both parties represent distinct views, most possibly having both passionate critics and supporters within the parliamentary chambers. The difference in speech length was not a factor here as the focus is on the micro-analysis of one EM.

### 4.3 Perception-based analysis of EMs: movement dynamics as affective qualities

When considering the speeches in their unfolding entirety, we see that they have an overall characteristic flow and dynamic. The micro-analysis focuses on moments of high affective engagement that stand out in particular. The multimodal EMs that surround them serve as units of analysis for the investigation of verbal and audiovisual multimodality.

#### 4.3.1 First impressions of the Bundestag speech

The German speaker, Katrin Göring Eckardt (KAGE), is a very enthusiastic and engaged speaker, and she speaks with a constant high volume and pitch. Her speech is interrupted by various (harsh) heckling from the audience which she mostly ignores. At one point she is interrupted by the president of the *Bundestag* (*Bundestagspräsident*) Wolfang Schäuble because of a speaking request by another politician which she allows. KAGE uses a lot of discursive beating gestures. Her overall speaking style is very clear, sometimes—by pronouncing syllable after syllable—it is highly emphasized. Figure 4 shows the unfolding of affective peaks along the speech.

Overall the speech is characterized by a rather moderate affective dynamics. Most of the 23 peaks are moderate (in gray), and only five show high affective dynamics (in red). The movement dynamics of the affective peaks in general are rather calm, concentrated, and determined. The green vitality contours indicate the movement dynamics of the EMs that surround the affective peaks.

#### 4.3.2 Affective dynamics: an expressive movement in the Bundestag speech

The affective dynamics described in this section concerns the last EM that shows high affective engagement in KAGE speech (EM 4, min. 14). It includes the audience’s reactions, as we take the perspective of the viewers that follow the speeches on their media devices. The description follows the eMAEX system (Kappelhoff et al., 2016). We use musical terms and the vocabulary for vitality forms as suggested by Stern (2010).

*In terms of its overall movement dynamics, the expressive movement is characterized by a steady crescendo that evolves into a peak phase with an enduring insisting character that terminates with a decrescendo including applause from the audience. This intense dynamics of the expressive movement begins rather quietly but then increases in a steady and pushing manner with increasing tension in voice and body. Speaking quickly and with a clear pronunciation the crescendo phase unfolds. During the peak phase, KAGE speaks with strong intonation and with a loud and tensed voice. While her gaze addresses the audience seated in front of her (this is where the Green Party members and the Christian Democrats are seated), she points repeatedly to the right in an accusing manner at the representatives from the right-wing party (Alternative für Deutschland, AfD). The rhythm of the peak phase includes significant pauses and fades away with a closing sentence that is drowned out by applause.*

#### 4.3.3 First impressions of the Sejm speech

**Figure 7:**
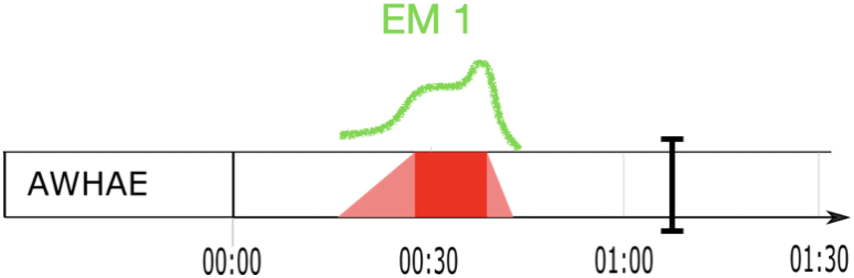
Sejm speech: affective peaks along the speech (only high affective engagement in red).

Dobromir Sośnierz (DOSO) is an extremely expressive speaker. His speech is characterized by immense emotionality, which is manifested both in his way of speaking and in his gestures. In his statement, DOSO presents his vision of the budget. The vocabulary he uses is very direct, sometimes even aggressive. DOSO directly addresses left-wing MPs, politicians from Law and Justice, and one specific MP, accusing them of lacking understanding of how the budget works. His bodily behavior also reflects his high affective involvement. We can observe full-body enactment, including leaning toward the audience in front of him and to his left and right-hand sides. DOSO performs numerous gestures, predominantly discursive beats. Some of them are performed with arms extended at the elbow. This affective dynamics shows in the visualization of affective peaks: in particular, almost his entire speech is one area with high affective engagement, embedded within one EM (Figure 7). Although the fact that DOSO’s speech is rather short certainly plays a role, it is still remarkable that he maintains a high affective engagement all the way through his speech.

The area with high affective engagement in DOSO’s utterance is highly dynamic, featuring heightened volume and pitch, sweeping upper body movements, expansive gestures, rhythmic beats, and animated facial expressions. During DOSO’s speech, individual voices of parliamentarians can be heard. It seems that these voices neither influence DOSO’s speech nor constitute comments on his statements.

#### 4.3.4 Affective dynamics: an expressive movement in the Sejm speech

The EM in DOSO’s speech is extremely dynamic and highly affective. Here, the area with high affective engagement is co-extensive with the EM:

*The Expressive Movement starts rather calmly but quickly develops toward a rapid increase in intensity, marking the start of the peak phase: after continuously accelerating speech pace, gestural beats increase in size and space. Successive, staccato gestural beats follow along with an explosive peak with even bigger and more energetic, repetitive beats and a change in pitch quality. This is followed by a slight decrease in intensity.*

#### 4.3.5 Summary and discussion

The perception-based descriptive analysis of the affective dynamics reveals that in both speeches affectivity unfolds in rhythmic patterns, sometimes variably and with changing affective intensity; in short speeches, high affective dynamics may even shape the entire speech. Visualizations and descriptions of the affective qualities reveal perceptive gestalts which are considered multimodal Expressive Movements. These EMs unfold as characteristic forms of vitality. Perception refers here to the perception of the speech as an audiovisual recording. Researchers, like viewers, can only perceive what the shot size and angle, the montage, and the recorded sound allow them to hear and see. Particularly relevant here is the role of the sound settings of the microphones, and whether they are set to strengthen the speaker’s voice and background or suppress audience reactions or not. The multimodal EMs described here thus emerge as multidimensional experiential gestalts in the process of watching the speeches on TV or on some kind of screen.

### 4.4 Acoustic-phonetic analysis of EMs

The instrumental analysis of the prosodic characteristics of the speeches was carried out as an exploratory study to investigate whether they would reveal similar forms of affective dynamics and thus support the descriptive analysis of EMs and their specific forms of affective dynamics. The subject of analysis is the sound of the recording, not the ‘real’ local speech sound happening *in situ*. This means also the acoustic-phonetic analysis also takes the perspective of the viewer and listener of the recorded speech.

The acoustic-phonetic analysis included the following variables: speech rate, vocalic and consonantal nPVI, syllable duration difference slope and intercept for interpausal time groups, intensity, and pitch frequency.

#### 4.4.1 The speech from the Bundestag

As KAGE’s speech is rather long and contains several moments showing higher affective dynamics, a moving time window approach was selected that zoomed in on the region within the speech that entailed the EM (4) described above.

**Figure 8:**
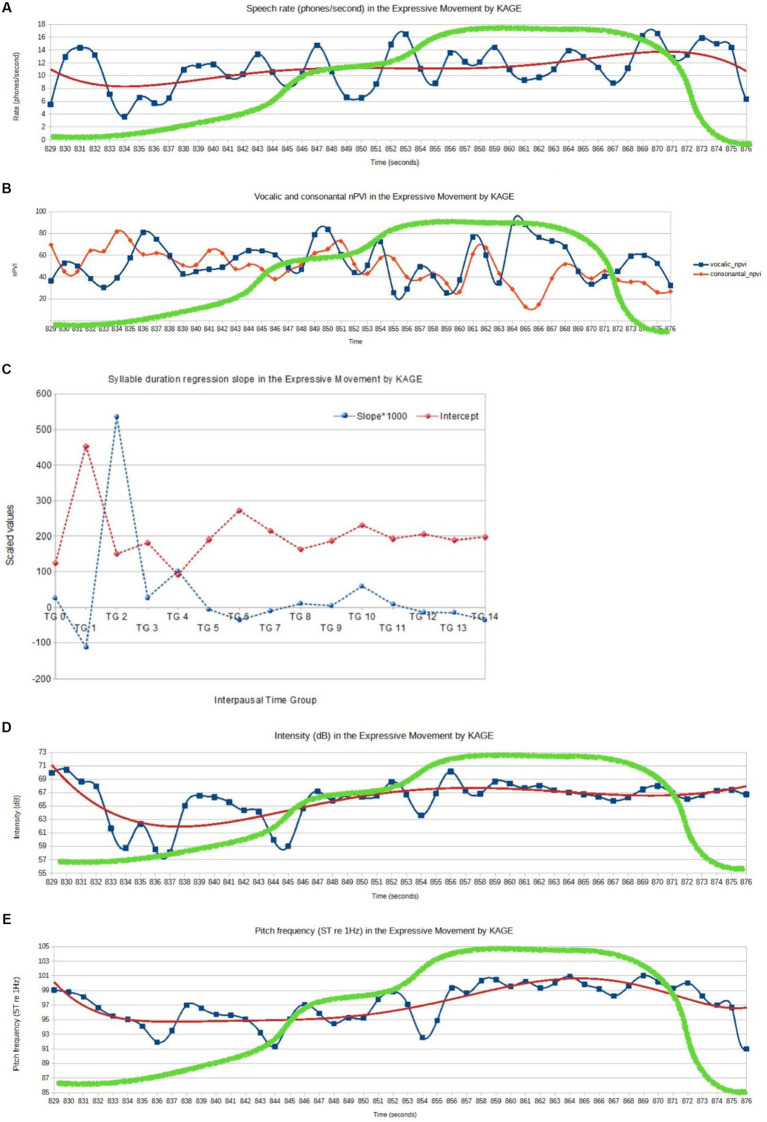
Acoustic-phonetic features of the speech and superimposed vitality graph (geen) from the descriptive analysis, within the range of the EM (KAGE). **(A)** KAGE, EM: speech rate (phone/second). **(B)** KAGE, EM: vocalic and consonantal nPVI (window size = 2 s, time step = 1 s). **(C)** KAGE, EM: mean values of syllable duration difference slope and intercept for interpausal time groups (TG stands for time group). **(D)** KAGE, EM: intensity (dB, window size = 2 s, time step = 1 s). **(E)** KAGE, EM: pitch frequency (semitones re 1 Hz).

The affective dynamics on the level of the acoustic-phonetic features of the speech, within the range of the EM, is displayed in Figure 8. It visualizes the variability of selected timing and pitch frequency parameters in the utterances of KAGE. The plots in Figure 8 represent the mean values for moving time windows within the EM range and refer to the *changes in the speech rate* (phones per second), *normalized Pairwise* Var*iability Indices* (*nPVIs*)*, intensity* (dB), and *pitch frequency* (semitones re 1 Hz) (last 3 s are not shown in the graphs as they contain no speech). The points are joined with a cubic spline curve. Additionally, the fifth-degree polynomial regression lines (red) are added to the first three figures to highlight more general, long-term tendencies. Figure 8C illustrates the values of *syllable duration* difference regression slope (the blue plot) and intercept (the red plot) for 15 *interpausal time groups* within the realization of the EM. In all figures, the green graphs show the affective dynamics as vitality contour from the descriptive analysis of the EM above. The green line was manually superimposed on the software-generated graphics.

In particular, the dynamics of the prosodic features within the EM differ from what is observed within the baseline dynamics of the speech. The differences do not occur only between central tendency or variability measures but also between the (complex) patterns of their changes over time. Moreover, as shown below, they are of different nature for different prosodic features, and they do not necessarily occur in all of them simultaneously. The speech rate within the EM [mean rate = 10.87 phones/s, SD (standard deviation) = 3.89] is lower than the baseline (mean rate = 11.99 phones/s, SD = 2.69), but the higher standard deviation value may indicate more dynamics in the changes of speech rate within the EM. The value of the vocalic nPVI within the EM is lower than the baseline (mean nPVI = 61.57, SD = 14.16 vs. mean nPVI = 55.08, SD = 14.16) which may mean a higher rhythmic stability, perhaps due to the strong, emphatic rhythm of speech. In the TGA, the mean of slope values for the EM (mean slope = 0.0362) is higher than the baseline (mean slope = −0.0099), indicating the tendency toward deceleration within the EM. The mean intercept value for EM (mean intercept = 202.68) was higher than the baseline (mean intercept = 198.63), which indicates a slightly slower average tempo and longer syllable durations in the EM. Speech rate and vocalic nPVI curves are complex, and their correlation with the vitality curve is not obvious. However, one may notice that the affective peak is preceded and ended with a speech rate of especially high ranges while the vocalic nPVI seems not only to gain more fluctuations after a short period of stability but the fluctuations show more periodic regularity.

In addition, the intensity is higher within the EM (mean intensity = 66.10 dB, SD = 5.41) than the baseline value (mean intensity = 65.20 dB, SD = 6.26). In this case, the lower standard deviation, corresponding to “flattened” dynamics, may be a result of an attempt to keep the voice louder all the time. As mentioned, however, it may be also due to compression used in voice amplification. The pitch frequency in the baseline is *ca*. 0.5 ST higher than the mean for the entire EM (97.78 ST with SD = 2.81 vs. 97.22 ST with SD = 3.08), but, as in the case of speech rate, a higher value of standard deviation indicates a higher spread of the values, i.e., operating on a wider range of pitch levels.

Both the intensity and the pitch frequency changes, in spite of high variability, follow the general shape of the vitality curve, increasing toward the affective peak and remaining at a high-level plateau almost till its end. As shown in the measures discussed above and in respective figures, the prosodic means of expression used by KAGE are complex and vary in the unfolding of the EM. The most remarkable feature is the increase in pitch frequency, highly correlated with intensity changes (Figures 8D,E), including the compression of the intensity curve in the affective peak region. Timing-related values, on the contrary, may suggest more prominent and regular fluctuations in this area, with a slight tendency toward deceleration.

Due to the relatively long duration of the EM and its complex internal structure, global measures of prosodic parameters may not fully reflect its dynamic nature. They may, however, be more evident for the affective peak when contrasted with the baseline characterized by the overall dynamics of the speech. The mean values of speech rate, intensity, and pitch frequency within the affective peak turned out to be higher than in their mean values within the entire EM as well as the baseline values (mean speech rate = 12.71 phones/s, SD = 2.58; mean intensity = 66.97 dB, SD = 4.67; mean pitch 99.51 ST, SD = 3.19) while the mean vocalic nPVI was still lower than the baseline value (mean nPVI = 60.69, SD = 18.01), and the mean slope is higher than the baseline but still lower than the mean value for the affective peak (slope = 0.0096, intercept = 204.08).

To sum up, the affective peak of the EM under analysis is easily distinguishable and characterized by distinct prosody. While the dynamic changes of prosodic features, initially predominantly increasing (but not necessarily in a stable, steady fashion), and decreasing in the final stage (again, this process may be quite complex as well), are typical of the EM as a whole, in KAGE, pitch frequency and intensity reach the highest values with a more limited degree of variability (“flatter” sections of the curves in Figures 8D,E), becoming prosodic indicators of the affective peak.

#### 4.4.2 The speech from the Sejm

The speech from the Sejm is short and almost co-extensive with one EM that unfolds with a single affective peak (cf. Figure 7, section 4.1). Consequently, the instrumental analysis of the prosodic parameters extends over the entire speech. As in the German example, instrumental measures of selected prosodic parameters related to pitch and duration were taken and their values were confronted with the results of the perception-based analyses of the EM with its affective peak area.

**Figure 9:**
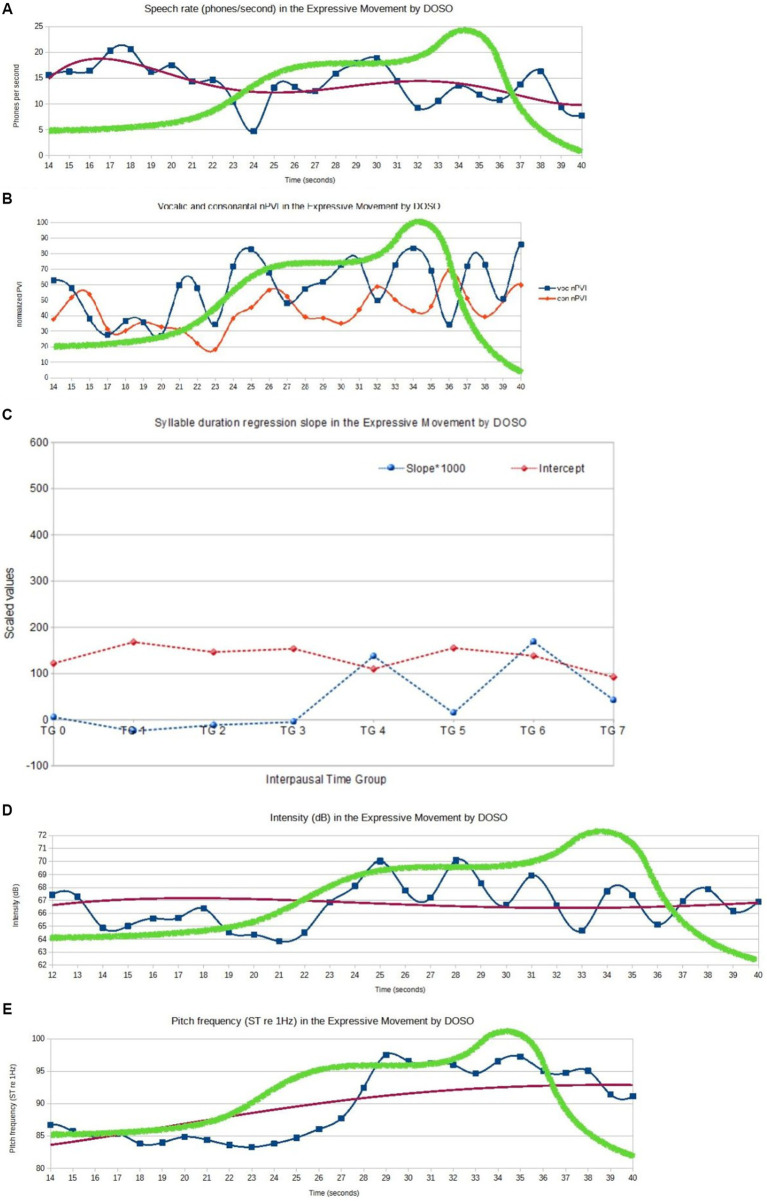
Acoustic-phonetic features of the speech and manually superimposed vitality graph (green), within the range of the EM (DOSO). **(A)** DOSO, EM: speech rate (SRMA, phones/second). **(B)** DOSO, EM: vocalic and consonantal nPVI. **(C)** DOSO, EM: mean values of syllable duration difference slope and intercept for interpausal time groups (TG stands for time group). **(D)** DOSO, EM: intensity (dB). **(E)** DOSO, EM: pitch frequency (in ST).

DOSO achieves extremely high levels of prosodic prominence in the affective peak, but the entire realization of EM is characterized by dynamic changes in prosody. In Figure 9, the mean values calculated from a moving time window within the EM are represented for speech rate (phones per second), vocalic and consonantal normalized Pairwise Variability Indices (nPVIs), intensity (dB), and pitch frequency (in semitones re 1 Hz). A fifth-level regression line is added (red) to indicate long-term tendencies. Figure 9C represents the results of the Time Group Analysis, i.e., syllable duration difference regression slope (the blue plot) and intercept (the red plot). While the speech rate varies throughout the realization of EM, with a decreasing tendency, its mean value remains very close to the baseline (mean rate 13.92 phones/s, SD = 3.80 vs. mean rate = 13.99, SD = 3.82). The mean vocalic nPVI is higher in the EM than the baseline, with an even more noticeable difference between the standard deviation values (voc.nPVI = 58.06, SD = 17.79 vs. voc.nPVI = 53.65, SD = 12.00), indicating a higher spread of the values. Time Group Analysis indicates that more duration regression slope variability occurs closer to the end of the EM, exhibiting more tempo differentiation in the final part of the EM. The mean intercept value for the EM is higher than for the baseline (intercept = 139.08 vs. intercept = 135.54). The values of the duration-related variables suggest that in the initial phase of the EM realization, the speaker has a decreasing tendency while closer to the affective peak, there are strong rhythm fluctuations. The periodic changes of the nPVI values become remarkable immediately before the first rise of the vitality curve, but they do not stop immediately after the affective peak. The mean intensity in the EM is very close to the baseline (66 dB, SD = 1.7 dB vs. 65.7 dB, SD = 2.04 dB). As in the case of the German speaker, it may be partially caused by compression but DOSO seems to speak at a high-intensity level from the very beginning of his speech. On the other hand, the difference between both the mean pitch value and its standard deviation in the EM and the baseline is striking (89.85ST re 1 Hz, SD = 5.45ST vs. 83.73ST, SD = 1.06ST). The mean pitch almost continuously rises until it reaches a plateau within the area of affective peak, with another peak after the vitality curve is steeply falling down. The relationship between pitch frequency and intensity is not as obvious here as in the German speaker. Intensity seems to initiate rapid prosodic changes just before the occurrence of the affective peak, with the pitch value changes following shortly afterward. The fluctuations typical of the intensity values within the affective peak got weaker gradually after the vitality line abruptly headed downward, while the pitch values gradually fell down as well with a certain delay.

The pitch change seems to be the dominant means of expressivity used by DOSO in his speech. It rapidly rises and remains higher throughout the EM to fall only at its end. Duration-related measures do not bring evident differences. They may require a quantitative exploration of a larger dataset to find more subtle tendencies.

The affective peak shows even more stable prosodic characteristics than in the German speaker. Pitch height reaches a plateau at a very high level (mean pitch = 95.13ST, SD = 1.55), and speech rhythm becomes regular and prominent, but the nPVI values (voc.nPVI = 63.44, SD = 14.64) grow probably due to the forced adjustment of the segmental durations to the strong rhythmic pattern. Similarly, the results of the TGA indicate significant changes in the second part of the EM. However, the speech rate is preserved, and not very different from the baseline value (speech rate = 13.56, SD = 3.05).

Similarly to what was observed in the German speaker, the area of the EM is characterized by more prosodic variability than the baseline. However, it is worth mentioning that the changes do not simultaneously occur in all the variables. Some of them, like the speech rate in DOSO, may remain almost unchanged or change in a way that cannot be properly captured by global measures like the mean or the standard deviation. In DOSO, extremely high pitch frequency remains the most prominent component of the affective peak prosody.

#### 4.4.3 Summary and discussion of the two speakers

The German and Polish speakers used for the demonstration of the present framework of analysis differ in gender, their political views, their way of speaking, and the duration of their EMs under analysis vividly differ as well (*ca*. 50 s for the German and *ca*. 26 s for the Polish speaker). Although both of them are fluent and expressive speakers, their “prosodic profiles” are very different. The speech rate within the EM in DOSO gradually decreases, while in KAGE the overall tendency is increasing. What they share, however, is strong fluctuations that seem to reflect perceivable peculiar rhythmic patterns. However, speech rate seems to be a secondary resource for emotional expression for the recordings under analysis. In both speakers, the initial part of the EM is characterized by lower fluctuations of nPVI (both vocalic and consonantal). This may suggest that the rhythm of speech becomes more prominent later in the EM, especially in the affective peak. Speech acceleration and deceleration phenomena were observed in both speakers. However, again, they manifested different strategies with regard to the variability of duration difference regression slope that corresponds to the average rate of duration change in the data. While in the realizations of the EM by KAGE more differentiation is visible at the beginning of the EM, for DOSO, larger differences between the subsequent time groups were found closer to the end of the EM. In the case of DOSO, the slope changes appear to quite strongly coincide with other prosodic features and subjective judgments of the speaker’s affective involvement, whereas for KAGE, this relationship is not obvious. Intensity also changes in different ways in the two speakers. In DOSO, it remains on a relatively low level, and only at the beginning of the affective peak, it abruptly grows, and later, within the affective peak, it strongly fluctuates, while in KAGE more fluctuations can be seen at the beginning of the EM, and later, she seems to operate on a higher level, with a more stable intensity values. Finally, the usage of pitch is clearly different in the initial part of the EM, where DOSO speaks with a relatively low voice and with limited pitch fluctuations, while the fluctuations in KAGE are much deeper than within the affective peak area. DOSO’s pitch frequency reaches a very high level in the affective peak, forming a “plateau” and falling down after the end of the affective peak. The pitch frequency values in KAGE’s affective peak also seem to be “compressed” and more stable, in this case, than at the beginning of the EM.

In many respects, different in the German and Polish speakers, especially referring to the temporal development of the EM, what is particularly important is that the dynamics of the prosodic features under scrutiny are? The above-mentioned changes in pitch in the section before the affective peak may be one example: in KAGE, pitch frequency changes are “dynamic,” while in DOSO, the pitch trace is flatter, with relatively low pitch frequency values.

While the dimensions of prosody are not fully independent, it should be noticed that the prosodic features potentially typically related to the expression of emotionality seem to be used selectively by both speakers. In KAGE, for example, pitch frequency and intensity highly correlate, but speech rate changes have clearly different dynamics. In DOSO, the similarity of pitch frequency and intensity traces is less obvious, and duration-related variables develop in a different way. Combining certain subsets of such features may be partially due to the physiology of speaking, to the properties of a given language, but can also be a part of the more or less conscious expressive strategy. Full analysis of their contributions and interactions will be subject to future work as it goes far beyond the scope of the present study.

What the results indicate, however, is that there is a marked difference in the acoustic-phonetic level between a baseline of the speech and the EM, which is particularly apparent in the affective peaks. The domains of pitch and intensity seem to be used for expressing emotionality more extensively by both the speakers. Pitch frequency and intensity reach higher values and seem to be compressed, with plateaus in the region of the affective peak. Duration-related measures provide evidence of a different kind, showing stronger periodic fluctuation. The instrumental analysis thus supports the perception-based recognition of EMs as perceptive gestalts.

### 4.5 Gesture analysis and audiovisual staging: complexity, visibility, and gestures as visible actions

This section considers the gesture performance as another significant facet of the multimodal orchestration of EMs with high affective dynamics in the parliamentary speeches. It begins with a general description of the multimodal orchestration and then addresses pertinent aspects of the respective performances more systematically and in more detail: in particular, complexity, visibility, and gestures as visible actions.

#### 4.5.1 Bundestag: multimodal orchestration of the EM

The EM unfolds around interjections from members of the right-wing party AfD who react to the warning of KAGE that the collaboration of AfD and the Christian Democratic Party CDU would be highly dangerous for democracy. KAGE then describes the members of the right-wing party metaphorically as ‘arsonists’ who subvert and destroy democracy and therefore need to be stopped by the ‘democrats’. The interjections from the right-wing MPs in the audience work as a boost for the affective dynamics of the speech. KAGE begins her speech in a calm and concentrated voice and subtle hand movements transforming increasingly in higher volume, speech velocity, and large gestures. Pragmatically, the EM begins with a warning but then changes to an accusation and a defense culminating in a clear positioning of KAGE as part of the ‘democrats’. This change in pragmatic meaning-making concerns both the verbal and the gestural part of the multimodal utterance.

Whereas the hand form in the beginning is rather non-specific and the movement is a subtle trembling, they change to a complex sequence of highly articulate specific gestural forms, with a shift to increased volume and speech rate. Concurrently KAGE makes a precision grip and an index finger pointing. For everyone to see, they are positioned in the center of the gesture space and are moved up and down in accentuated and rhythmical beats.

In the moment of highest affective intensity, the speaker shifts her upper body and gestures toward the right side of the audience (where the right-wing party has their seats). With this change in body orientation, the gesture space moves to the right too, the MPs from the AfD concurrently become the addressees of repeated index finger pointings and a decisive/determined negating sweeping away gesture. With the very last phrase of the last gesture unit, KAGE embodies a gestural full stop: with an emphatic downward beat, performed with a both-handed pointing gesture she marks the end of the argument and the strong stance she is taking against the interferers.

In a nutshell, KAGE’s change of affective dynamics within the EM is not only traceable on the level of the speech performance but also in regard to the communicative actions and the movement qualities of the hand gestures and body posture.

##### 4.5.1.1 Gestural complexity

**Figure 10:**
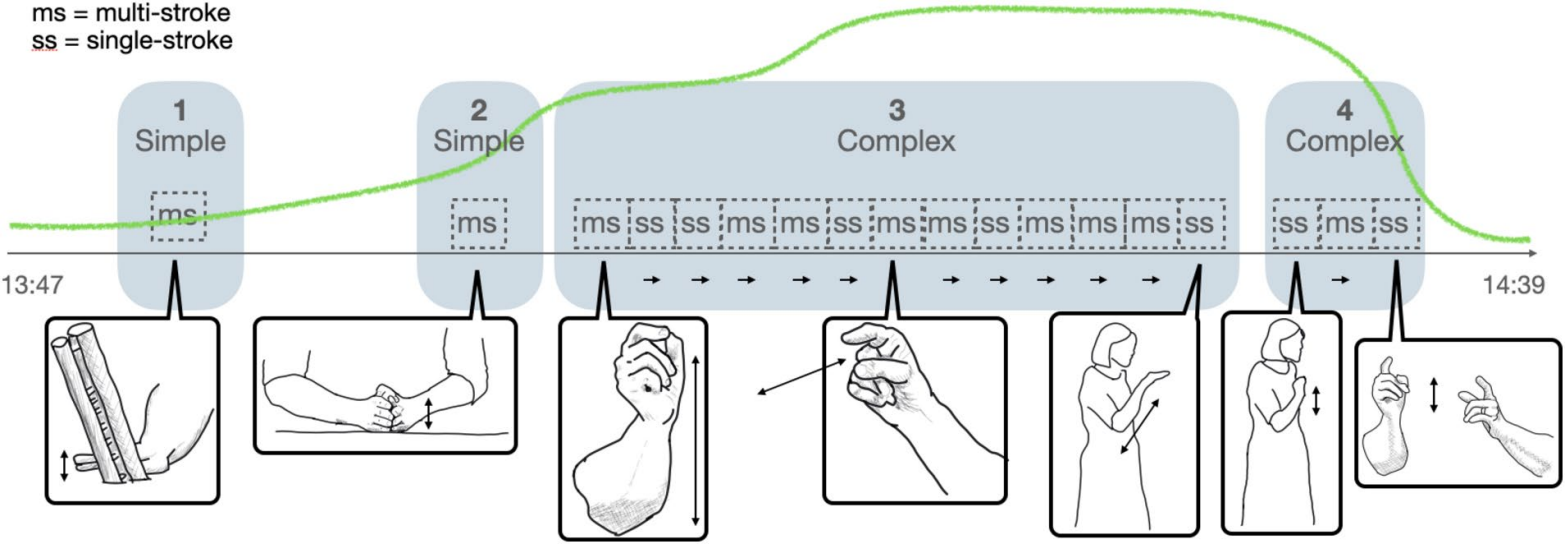
KAGE, the complexity of gestural performance.

Within the approximately 1-min-long EM, KAGE produces a series of four gesture units (Kendon, 2004, chapter 7) that increase in complexity and visibility over time. The increase in complexity concerns the internal structures of the gestural units. While the first two gesture units in the EM are simple units with only one gesture phrase (Kendon, 2004, chapter 7), the following two gesture units consist of 13 and 3 gesture phrases (Figure 10).

**Figure 11:**
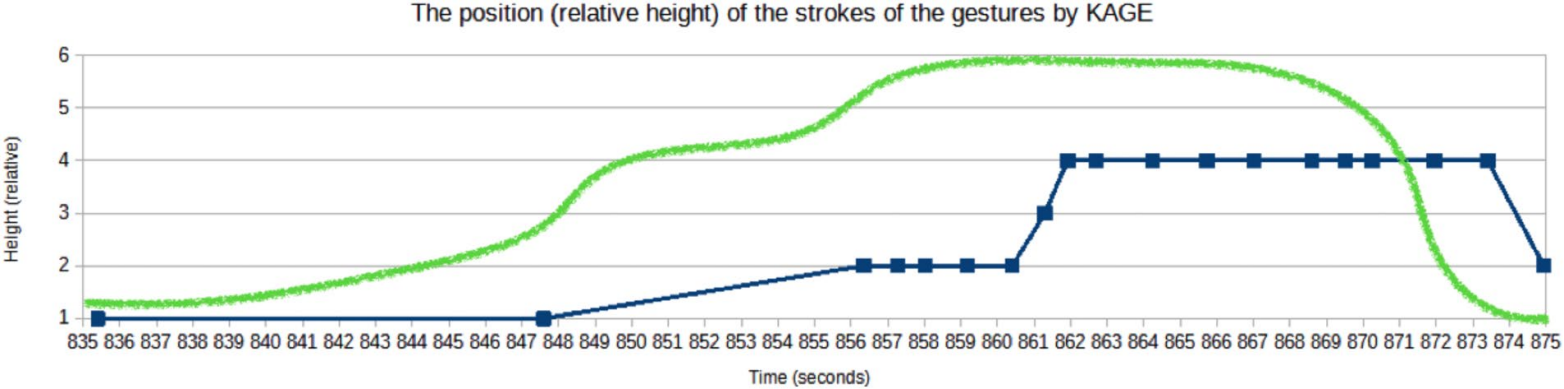
The vertical coordinate of the position of stroke realization by KAGE within the range of the EM (where “1” is the height of the abdomen and “6” is above the head) (Peak: from 861st to 874th sec).

**Figure 12:**
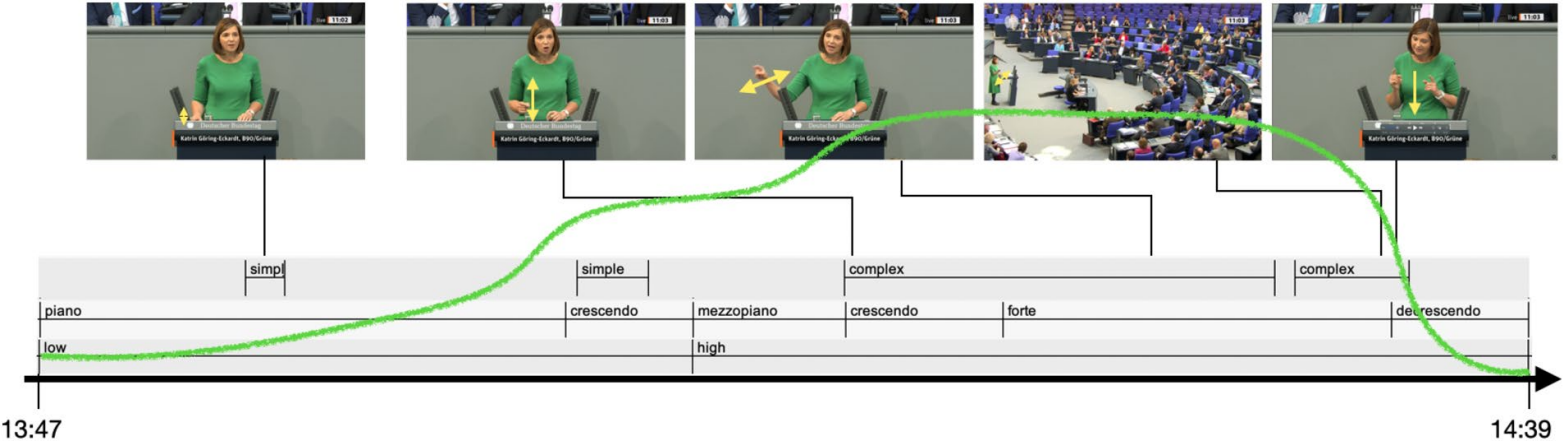
KAGE, vitality contour, musical terms, and the visibility of gestures in the image space as the EM unfolds. Recordings from parliamentary debates are sourced from Deutscher Bundestag.

Simple Unit (1) is formed by one multi-stroke phrase (Figure 10): KAGE makes small rhythmical beats on the rostrum that go along with speech rhythm and accentuation. Simple Unit (2) evolves as one multi-stroke phrase, here KAGE’s gesturing changes to subtle, vibrating up–down movements remaining on the rostrum, anchored at the wrist of her right hand. With the third gesture unit, KAGE reaches the affective peak phase, which unfolds as a highly complex gesture unit (3). Alternating between multi-stroke and single-stroke phrases, a sequence of 13 gesture phrases unfolds. KAGE makes a series of rhythmical tappings on the rostrum and then changes to accentuated pointing beatings toward the right side of the parliament. Over this sequence she moves her gestures higher up in the gesture space, from the lower right to the center and eventually to the right periphery, concurrently using her arm to perform the accusing pointing movements (Figures 11, 12).

##### 4.5.1.2 Visibility of gestures and audiovisual orchestration

The increase in gestural complexity goes along with the higher visibility of the gestures. During the affective peak, KAGE not only moves her hands up to the center of the gesture space, but they concurrently also become larger. Figure 11 visualizes the vertical coordinate of the stroke positions, showing a similar rising and falling pattern as the complexity of gesture units. This gestural performance makes the hands more visible not only for the attending audience in the parliament but also for the viewers on the screens, as the mise-en-scène of the audiovisual image places KAGE in the center of the image space and a medium shot perspective for the most part of the EM.

The gesture units unfold an affective dynamics that can be described as a flow from crescendo (units 1 and 2) to crescendo and forte (unit 3) to decrescendo (unit 4) (Figure 12). Gestural complexity *goes along with* the high affective dynamics of the expressive movement. However, while her gestures are clearly becoming more visible to the audience in the parliament, this is not the case for the viewers of the audiovisual recording. Because a cut to a wide shot reduces the visibility of the gestures dramatically making a determined sweeping away gesture along with its expressive dynamics nearly invisible (Bressem and Müller, 2014) (Figure 12). The insisting prosodic qualities (pitch, intensity), however, remain perceivable for them. Then, with a camera-shift back to a medium shot there is an increase in visibility for the spectators at home, concurrently showing a series of single-, multi-, single-stroke phrase, e.g., a decrease in internal complexity. This last gesture unit then contributes significantly to the termination of this EM: KAGE makes a kind of gestural exclamation mark, a highly accentuated, energetic both-handed downward pointing gesture (Figures 11, 12).

Thus, over the course of the unfolding of the EM, the gestural movements not only become more complex but also more visible: they become bigger, are moved higher up in the gesture space, and are shifted to the center of the image space (Figure 12). The visibility for the spectators changes when the camera perspective changes to a wide shot. This zooming away from the speaker’s gestures decreases the perceivable expressivity of the gestural performance for the spectators of the televised performance. However, the audiovisual perspective shifts concurrently include the MPs and the accused audience, thus creating a visual image that foregrounds the interaction between the speaker and parliament.

Summing up, a closer look at the sequence of four gesture units shows a steadily rising affective dynamics materializing as increasing and decreasing complexity and visibility of gestural movements. Dynamic changes in gestural complexity participate in the multimodal orchestration of this area with high affective engagement. They are one facet of the multimodal orchestration of affectivity as stance. For the audience that follows such televised speeches, the audiovisual orchestration contributes significantly to the multimodal orchestration of an affective stance-taking.

#### 4.5.2 Sejm: multimodal orchestration of the EM

**Figure 13:**
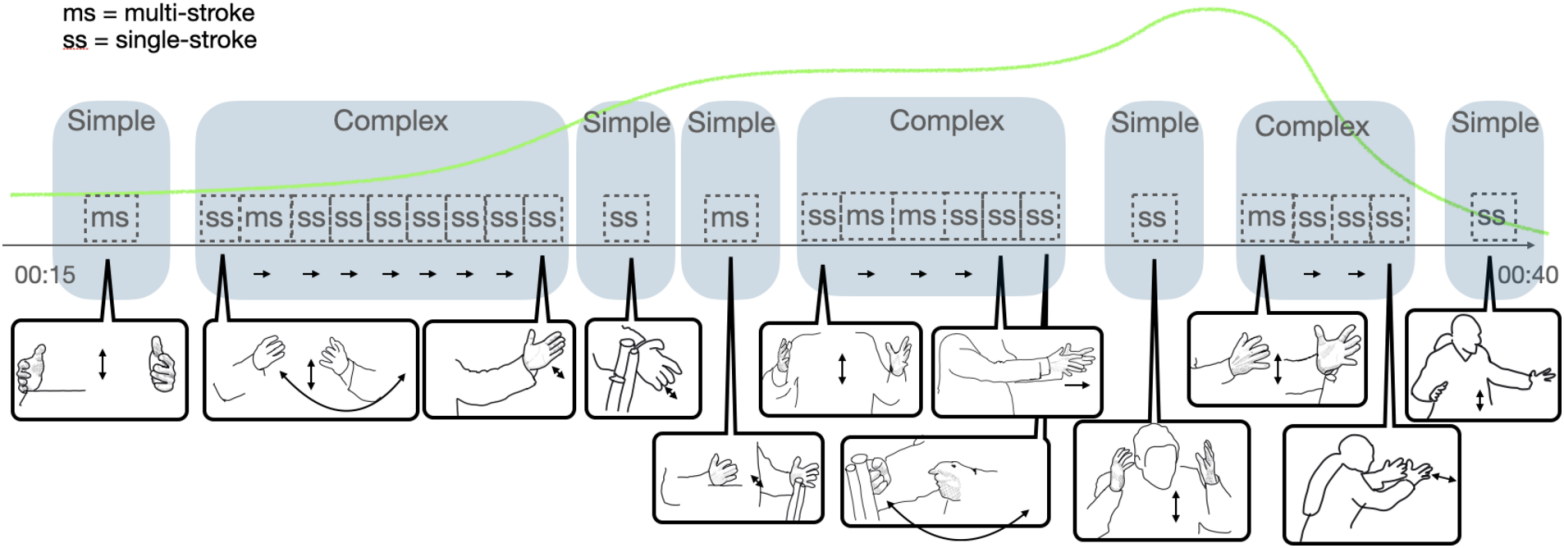
DOSO, the complexity of gestural performance.

**Figure 14:**
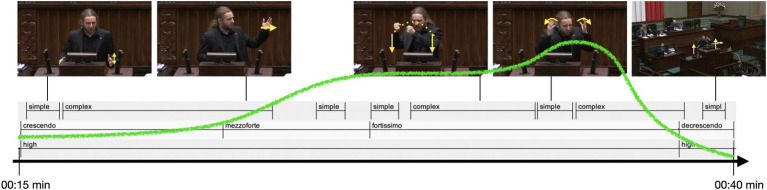
DOSO, vitality contour, musical terms, and the visibility of gestures in the image space as the EM unfolds. Recordings from parliamentary debates are sourced from System Informacyjny Sejmu.

The EM begins with a rhetorical question addressed to all the members of parliament, in which DOSO expresses his doubt whether they understand the essence of budgetary policy which relies on taking goods from some people in order to give them to the others. At this stage, at the onset of the EM, the pitch is slightly rising and speech is starting to gain more rhythmicity due to regular changes in intensity and pitch frequency. The speaker’s engagement is gradually intensifying. He is making several beat gestures with both hands. These are short, energetic movements initially performed just above the rostrum, and then higher and higher (Figures 13, 14). The final beat in this part of the utterance is executed at the upper chest level. This part of the speech can be described as a crescendo (Figure 14). Then, DOSO directly addresses one of the members of parliament. During this part of the speech, he only makes two gestures. One pointing straight at the MP to whom the speech refers. The stroke phase in the described pointing gesture is executed at the head level. During the gesture, DOSO turns toward the MP and looks in their direction. The second gesture is a depictive (referential) gesture (Müller, 2024) embodying aspects of DOSO’s spoken utterance. This part of the speech can be characterized as mezzoforte. Suddenly, an affective peak in the speech occurs, lasting approximately 11 s, during which 12 gesture phrases are executed. Most of them are quick, performed with both hands, with a huge energy load in the beats, and high up in the gesture space.

Approximately halfway through this part of the speech, DOSO makes another depictive (referential) gesture. It enacts the action of giving and taking. Both hands performing the gesture are straightened at the elbow and mimic the transfer of an object from the left side of the speaker to the right side (Figure 14). This fortissimo part of the speech can be relatively easily identified on the basis of prosody itself. Pitch frequency remains on a distinctively high level, intensity is fluctuating, as well as the vocalic nPVI, making the rhythmic structure of this section even more prominent. Although clearly noticeable in earlier sections, speech-gesture synchrony is extremely vivid in the fortissimo section.

In the final part of the speech, described as a decrescendo, DOSO only performs beat gestures in the zone just above the rostrum (Figures 13, 14). His voice is gradually falling down to his baseline pitch frequency, pitch range is narrowing down. While intensity is not decreasing extremely, the voice may be perceived as slightly calming down and more stable.

##### 4.5.2.1 Gestural complexity

In DOSO’s speech, the dynamics of the EM also show in the complexity and visibility of the gestural performance—although the kind of complexity in which it unfolds is different from KAGE. DOSO is a very engaged gesturer, and he has very little time to speak. In fact, he gestures basically all the time along the unfolding of the Expressive Movement (approximately 25 s long). During this time, he produces a series of eight gesture units (Figure 13). The beginning of the EM evolves in a succession of a simple and a complex gesture unit, with the increasing affective dynamics (see the green vitality contour in Figure 13) we see a succession of five gesture units, the affective peak (fortissimo) unfolding as a sequence of complex, simple, complex units. The moment of high affective engagement terminates with a decrescendo and with the ending of complex and a simple gesture unit (Figures 13, 14). Most of the gestures realized within EM are single-stroke gestures (18 single-stroke gestures and 6 multistroke gestures).

In contrast to KAGE’s performance, DOSO’s EM does not show an increase in internal complexity. This indicates that there is not a simple match of high affective stance and complexity of gestural performance. This counters simplistic correlations of affective involvement with gestural performance, following the rule: the higher the affectivity, the higher the complexity of gestures. The multimodal orchestration of EMs is more variable than this.

##### 4.5.2.2 Visibility of gestures and audiovisual orchestration

**Figure 15:**
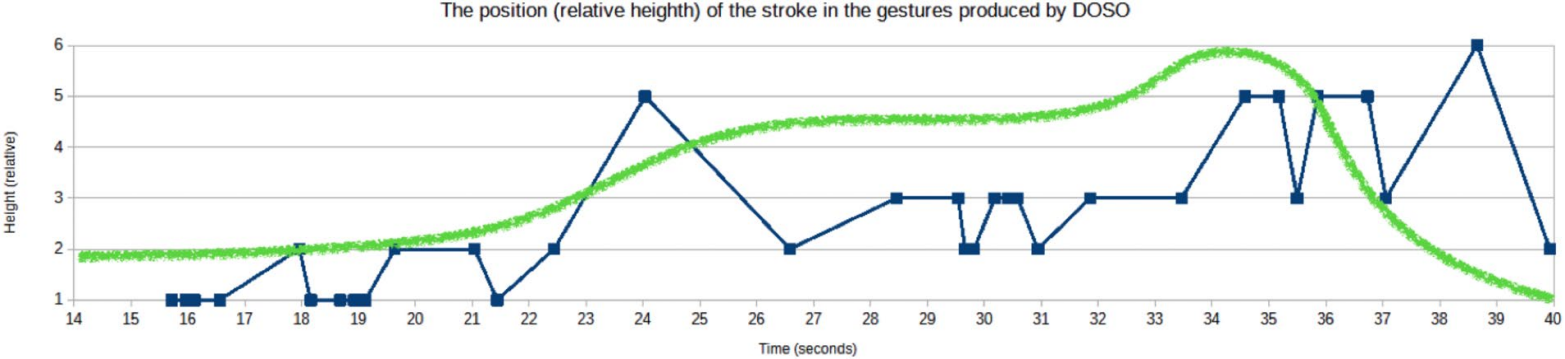
Vertical coordinate of the position of stroke realization (where “1” is the height of the abdomen and “6” is above the head).

On the other hand, when it comes to the visibility of gestures, we see that, similar to KAGE’s moment of high affectivity, DOSO moves his gestures increasingly higher up in the gesture space, thus moving them into the focus of joint visual attention (Figure 14). So, during the crescendo and mezzo-forte moments, most of the gestures are produced just over the rostrum, while in the peak phase, they are predominantly executed at the height of the upper chest area or higher. In short, moments of high affective dynamics appear to go along with a tendency to raise hands—also in DOSO’s speech. Figure 15 shows how the position of the stroke in the gesture space changes along with the vitality contour of the expressive movement.

The green vitality contour (superimposed manually) illustrates how also in DOSO’s EM different aspects of movement dynamics work together in forming this moment of high affective engagement (Figures 14, 15). We see a heightened visibility of the gestures and a change in movement dynamics of gestural and speech performance in particular in the affective peak phase.

We also see similar camera work in the Sejm as in the Bundestag recordings of the speeches. While most of the time, the speaker is placed in the center of the image space (with a medium shot), toward the end of the EM the camera perspective changes to a wide shot, reducing the size of the speaker for the spectators and hence the perceivable expressivity of the gestural performance. Altogether, the move *in camera*-perspective orchestrates the decrescendo phase of the EM.

#### 4.5.3 Summary

Micro-analyses of the gestural performance and its audiovisual staging have shown that moments of high affective engagement go along with an increased complexity in KAGE’s case and heightened visibility of gestural movements in both EMs. Gestures in high affective areas are performed in the upper part of the gesture space, which makes them more visible to the viewers on the screens and in the audience and increases their relevance as objects of joint attention. In areas with high affective engagement, gestures thus appear to become foci of joint visible attention. In both recordings, the placement of the gestures in the gesture space and the size of the gestures in relation to the image space is modulated by a change between medium shot and wide shot toward the end of the affective peak thus reducing visibility and hence the perceivable expressivity of the speaker’s gesturing, but also bringing the audience within the parliament into the ‘image’ and the attention of the spectators.

It is in this sense that audiovisual and verbal multimodality are intertwined and it is this integrated orchestration that modulates the affective perception of the spectators in their process of watching audiovisual documentations of political speeches.

## 5 Summary and discussion

The media-aesthetic framework proposed in this paper offers a perspective on multimodal affective stance-taking in specific media ecologies. Exemplified by analyses of parliamentary discourse, it addressed in particular the intertwining of audiovisual multimodality and the multimodality of speaking. It advocates a phenomenological approach to affective dynamics of audiovisual composition and multimodal debates because it is interested in how spectators are moved by the orchestration of televised debates. In order to do this, the concepts of expressive movement and vitality contour served as theoretical and methodological references.

### 5.1 EM and vitality contours as keys to analyzing affectivity as stance

Conceived as multidimensional experiential gestalts, multimodal EMs allow affectivity to be reconstructed as unfolding movement dynamics and offer a way to address the mobilization of affective stance as modulation of the perception of the viewers.

To illustrate this approach, a perception-based analysis of EM (movement dynamics as affective qualities) was combined with an instrumental analysis of speech prosody. This means two independent empirical approaches to identifying EMs as dynamic gestalts were applied. Instrumental measures of EMs did reveal similar characteristics of affective contours as in the subjective analysis of EMs.

In a micro-analysis of the temporal unfolding of the gestural performances within the EM, two aspects of the multimodal orchestration of areas with high affective engagement received particular analytic attention: the complexity and visibility of the gestural performance. These aspects were chosen because they appeared as pertinent characteristics of moments with a high level of affective engagement.

### 5.2 Multimodal orchestration of stance

The analysis of the audiovisual staging of the debates shows that, for example, in KAGE’s speech, the EMs that the viewers of parliamentary debates perceive on their screens are not only the multimodal enactments of a speaker but include audience interventions or the absence of them. The highly affective stance of KAGE reacts to interventions and shows that affective stance even in such a rather monologic context as a parliamentary speech is a joint process of affective dynamics within the parliament, which then is framed in a particular manner for the spectators (shift from medium to wide shot).

Here, KAGE formulates a complex stance of not only ‘being against’ but ‘being ready to fight against’ the actions of the right margins. She is performing a multimodal speech act of accusation by verbally accusing and intensely and repetitively pointing with a stretched index finger in the center of the gesture space while also gazing at the ones being accused. The peak ends in a change of camera perspective: concurrently the members of the parliament are shown in the shot. The mise-en-scene positions them at the top center showing their faces and their applause modulating a sense of interactive support and wideness, while the accused ones are visible on the lower side of the image space only from the back. The unfolding of the complex stance is therefore grounded affectively. It is exactly this basic form of human understanding, the immediate affective experience that is understood as stance.

### 5.3 Comparison of the two speeches

In the German recordings, these interactions with the audience are clearly audible, while this is different in the Polish material. In the case of the Polish parliamentary speeches, a lesser degree of interactivity between the speakers and the audience is visible and hearable. As mentioned above, this may be due to a few factors, starting from the strictly technical ones (e.g., microphones intentionally set to limit the hearability of the voices from the hall), or probably also to cultural ones. While during the speech of DOSO a few voices can be heard from the hall, they do not seem to influence his speech in an obvious way. On the other hand, one cannot say that there is no emotional tension between many of the listeners and the speaker, who is often perceived as radical and controversial.

The televised debates appear to differ in their multimodal orchestration of affectivity: whereas DOSO’s speech is shown as unfolding its affectivity, especially on the level of the speaker’s performance, KAGE’s speech is staged as a debate and includes audience interventions. Similar staging forms are observed in the material from the German Bundestag (cf. Kindler-Mathôt et al., 2025) but not in the Polish data, where noticeable interactions are very limited.

Further research will show whether this staging of the speeches as more monologic or more dialogic results from different cultural standards and/or whether these are part of a more general understanding of how parliamentary debates are conceived in these two cultures. Political science studies show that, for example, the architecture of parliaments can be an expression of a specific understanding of parliamentary work (Minkenberg, 2020). Clearly, the seating arrangements prefigure specific kinds of interactions.

What we see here is how, even in such ‘neutral’ media formats, an orchestration of political processes is realized. Affective dynamics such as rising tension, beating rhythms, forcefulness, or softness characterize them and shape the embodied perception of the spectators. In both speeches, affective stances are realized as dynamically unfolding in expressive movements. Such observations underline the relevance to studying the audiovisual portrayal of democratic processes and representation (Kindler-Mathôt et al., 2025).

## 6 Conclusion

This article has presented a media-aesthetic approach to analyzing the multimodality of speech in audiovisual media. We have argued that the empirical analysis of multimodal speech data appearing in audiovisual media requires a reflection and methodological consideration of their specific media ecologies. By starting from the physically perceptible unfolding of affective multidimensional forms of experience, so-called EMs and vitality contours, and not from a specific linguistic phenomenon, we have suggested a significant change of perspective. In accordance with Goodwin et al.’s (2012) notion of Emotion as Stance, we take the unfolding of affectivity as the anchor point to describe stance-taking not primarily on the level of single utterances nor only on the level of the spoken word but as emerging and unfolding affectivity orchestrated by both the multimodality of speaking (gesture and speech) and the audiovisual multimodality. Affective stance is not primarily bound to a specific gesture or a specific verbal expression; rather, it unfolds as a dynamic contour that emerges as a holistic gestalt from the multimodal performance of a speaker and the audiovisual staging of the speech. When we research affective stance-taking in audiovisual broadcasts of political speeches, we thus need to consider the expressive character of audiovisual compositions. It is the expressivity of the audiovisual staging that engages with the embodied perception of the viewers as an aesthetic affective experience. For the viewers of the broadcast speeches, the perception of the multimodal performance of speaking is framed by audiovisual multimodality. Against a media-aesthetic approach, virtual interaction is as embodied as face-to-face interaction. It is the multidimensional experiential gestalt of the televised speech and gesture performance that touches the viewers. In short, taking an affective stance in the context of political debates in the media must be considered as feeling an affective stance in the first place.

We believe that this media-aesthetic perspective may contribute to a better understanding of the power of audiovisual formats such as TikTok, YouTube, or Instagram Reels in the political arena (cf. Greifenstein et al., 2018; Kindler-Mathôt, 2025; Kindler-Mathôt et al., 2025; Müller, in press; Müller and Kappelhoff, 2018). It can provide a valid starting point for future comparative research of multimodal stance-taking, be it across cultures, discourse genres, or different forms of political debates.

## Data Availability

The original contributions presented in the study are included in the article/supplementary material, further inquiries can be directed to the corresponding author.

## Footnotes

^1^https://www.bundestag.de/mediathek?videoid=7387900#url=L21lZGlhdGhla292ZXJsYXk/dmlkZW9pZD03Mzg3OTAw&mod=mediathek [Accessed 12 July 2024].

^2^https://www.bundestag.de/mediathek?videoid=7387986#url=bWVkaWF0aGVrb3ZlcmxheT92aWRlb2lkPTczODc5ODY=&mod=mediathek [Accessed 12 July 2024].

^3^https://www.sejm.gov.pl/sejm9.nsf/transmisja.xsp?documentId=D3D275F09D241460C12584EA004331A0&symbol=WYPOWIEDZ_TRANSMISJA.
